# Programmed Hydrolysis in Designing Paclitaxel Prodrug for Nanocarrier Assembly

**DOI:** 10.1038/srep12023

**Published:** 2015-07-13

**Authors:** Q. Fu, Y. Wang, Y. Ma, D. Zhang, J. K. Fallon, X. Yang, D. Liu, Z. He, F. Liu

**Affiliations:** 1 School of Pharmacy, Shenyang Pharmaceutical University, Shenyang 110016, China; 2 Key Laboratory of Structure-Based Drug Design and Discovery, Ministry of Education, Shenyang Pharmaceutical University, Shenyang 110016, China; 3 Division of Molecular Pharmaceutics, Eshelman School of Pharmacy, University of North Carolina at Chapel Hill, Chapel Hill, NC 27599, USA; 4 School of Chinese Materia Medica, Guangzhou University of Chinese Medicine, Guangzhou 510006, China

## Abstract

Nanocarriers delivering prodrugs are a way of improving *in vivo* effectiveness and efficiency. For therapeutic efficacy, the prodrug must hydrolyze to its parent drug after administration. Based on the fact that the hydrolysis is impeded by steric hindrance and improved by sufficient polarity, in this study, we proposed the PTX-S-S-VE, the conjugation of paclitaxel (PTX) to vitamin E (VE) through a disulfide bridge. This conjugate possessed the following advantages: first, it can be encapsulated in the VE/VE_2_-PEG_2000_/water nanoemulsions because of favorable hydrophobic interactions; second, the nanoemulsions had a long blood circulation time; finally, the concentrated glutathione in the tumor microenvironment could cleave the disulfide bond to weaken the steric hindrance and increase the polarity, promoting the hydrolysis to PTX and increasing the anticancer activity. It was demonstrated *in vitro* that the hydrolysis of PTX-S-S-VE was enhanced and the cytotoxicity was increased. In addition, PTX-S-S-VE had greater anticancer activity against the KB-3-1 cell line tumor xenograft and the tumor size was smaller after the 4^th^ injection. The present result suggests a new way, use of reduction, to improve the *in vivo* anticancer activity of a prodrug for nanocarrier delivery by unshielding the ester bond and taking off the steric block.

A prodrug improves the properties of a parent drug by linking with or being modified by a functioning or non-functioning molecular group^1^,^2^. Prodrug strategy has been used to improve aqueous solubility, enhance chemical stability, promote transport across epithelial cells, reduce pre-systemic metabolism, and increase oral absorption^3^,^4^,^5^,^6^. It is administered in an inactive form, and it must convert or hydrolyze to its active form through physiological metabolism^7^,^8^. As estimated, prodrugs accounted for about 17.8% (34 of the 191) of new chemical entities approved between 2000 and 2008, and prodrug prevalence was 15.4% among the top 100 bestselling small molecular weight drugs in 2008^9^,^10^.

Paclitaxel (PTX) is widely used for the treatment of non-small cell lung cancer, breast cancer, ovarian cancer and AIDS-related Kaposi’s sarcoma^11^. Both Taxol^®^^12^,^13^ and Abraxane®^14^,^15^ are commercial PTX formulations approved by the FDA. However, due to their low drug loadings and the problems associated with the burst release, experts have applied a prodrug strategy to improve the *in vivo* performance of PTX^16^,^17^. Some researchers have conjugated PTX with a hydrophilic polymer^18^,^19^,^20^,^21^,^22^,^23^, but the drug loading was still very low. In addition, the half-lives of the formulations are too short, leading to poor efficacy *in vivo*, although this strategy has prolonged the release and exhibited promising anticancer activity *in vitro*. Others have conjugated PTX with a hydrophobic chain^24^,^25^,^26^,^27^, in the hope that it could improve the compatibility between cargo and carrier, facilitating encapsulation in a nanocarrier. Nevertheless, the potential anticancer activity was limited due to poor hydrolysis, as evidenced by the fact that no more than 10% of PTX-linoleic acid conjugate was hydrolyzed to PTX at 48 h^28^.

In our group, PTX-VE (Fig. 1) has been synthesized by conjugating PTX and vitamin E (VE) together via an ester bond, and it has been encapsulated in the VE/VE_2_-PEG_2000_/water nanoemulsions (NES) because PTX-VE, VE, and VE_2_-PEG_2000_ were molecularly matched. Based on this, we have codelivered PTX and 5-fluoroucacil in a same nanoparticle by their conjugation with the VE^29^. What is more, using the same strategy, theranostic (PTX, and sulforhodamine B) has been also achieved^30^. However, PTX-VE did not release active PTX *in vitro* within 48 h, resulted in negotiated cytotoxicity. Thus, the aim of this paper was to find out a substitute for PTX-VE. We initially demonstrated that its hydrolysis was impeded by steric hindrance and improved by sufficient polarity, by grafting different groups onto PTX. Then, we proposed and synthesized PTX-S-S-VE, PTX conjugated with VE via a disulfide bond, a redox sensitive bridge (Fig. 2). This bridge would be broken in tumor cells because of the sufficient glutathione (GSH) concentration (2–8 mM)^31^, to promote the hydrolysis. In addition, the PTX-S-S-VE can also be encapsulated and assembled as PTX-S-S-VE/VE/VE_2_-PEG_2000_/water NES for administration because of favourable hydrophobic interactions between the prodrug and the nanocarrier. The tissue distribution, *in vivo* antitumor activity and hemolytic activity were evaluated for PTX-S-S-VE/VE/VE_2_-PEG_2000_/water NES, with PTX-VE/VE/VE_2_-PEG_2000_/water NES and Taxol^®^ as reference formulations.

## Results and discussion

### Proof-of-concept

**Figure 3:**
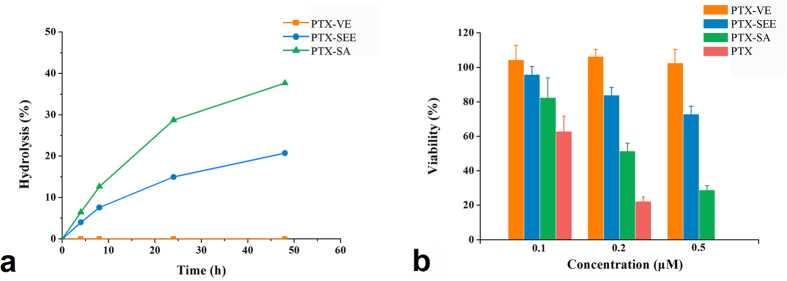
Hydrolytic release profiles in pH 7.4 PBS containing 7% DMSO and 0.1% Tween 80 (**a**) and *in vitro* cytotoxicity against KB-3-1 cell line for PTX-VE, PTX-SEE, and PTX-SA (**b**). These studies were carried out at 37 °C.

In the hydrolysis study, the PTX-VE was not hydrolyzed until 48 h. The reason for the poor hydrolysis of PTX-VE might be that the hydrolytic site (C-2' hydroxyl group in PTX) is sterically shielded by the VE. To test this hypothesis, PTX-SEE (the VE group substituted by a methyl group) was synthesized. Both PTX-VE and PTX-SEE are hydrophobic. As shown in Fig. 3a, approximately 20.7% of PTX-SEE was hydrolyzed to free PTX by 48 h, a much higher percentage than for PTX-VE. This indicates that steric hindrance could be a major reason for impeded hydrolysis.

In addition to this, Markovic *et al.* established a good correlation (R = 0.9924) between solvolytic rate constants and polarity, indicating enhanced polarity could improve hydration for a prodrug^32^. It is reasonable to assume that the hydrolysis rate is dependent on polarity to some extent. Therefore, we conjugated PTX and succinic acid directly to produce PTX-SA. In comparison with PTX-SEE, PTX-SA had increased polarity, which was evidenced by the retention time (6.7 min for PTX-SA and 31.7 min for PTX-SEE) in a reversed phase chromatography system (Figure S6). As expected, PTX-SA had a higher hydrolytic profile than PTX-SEE, suggesting that sufficient polarity might be the other reason for hydrolysis.

Therefore, decreased steric hindrance and enhanced polarity favor increased hydrolysis.

### Cytotoxicity demonstration

For PTX, the hydroxyl group at the C-2' position is important for its anticancer activity and it must therefore be exposed to the external environment to have its effect^16^,^17^. Thus, the hydrolytic rate for a PTX prodrug may therefore influence its *in vitro* cytotoxicity. In this research, the cytotoxicity was investigated on the KB-3-1 cell line using the MTS method. Free PTX, PTX-SA, PTX-SEE, and PTX-VE were added to the cells, respectively, with concentrations ranging from 0.1–0.5 μM. As shown in Fig. 3b, PTX-VE had no inhibitory effect when concentration was up to 0.5 μM because of the negligible hydrolysis, and its IC_50_ value was as high as 2.67 μM. For PTX-SEE, the inhibition effect was greater, and the viability was about 72.8% when cells treated with 0.5 μM of PTX-SEE. In the case of PTX-SA, the IC_50_ was 0.252 μM. In addition, free PTX had the strongest inhibition effect. The cytotoxicity did correlated well with the hydrolytic rate.

### Rational design for PTX-S-S-VE

In a previous study, we have developed PTX-VE/VE/VE_2_-PEG_2000_/water NES and demonstrated sufficiently high drug loading and improved pharmacokinetics, because the PTX-VE was subjected to strong interactions with the VE and VE_2_-PEG_2000_. However, the cytotoxicity of PTX-VE was very low because of poor hydrolysis. Therefore, in this study, we would like to develop a substitute for PTX-VE, by maintaining the VE group and changing the linker, in order to improve the hydrolysis.

**Figure 4:**
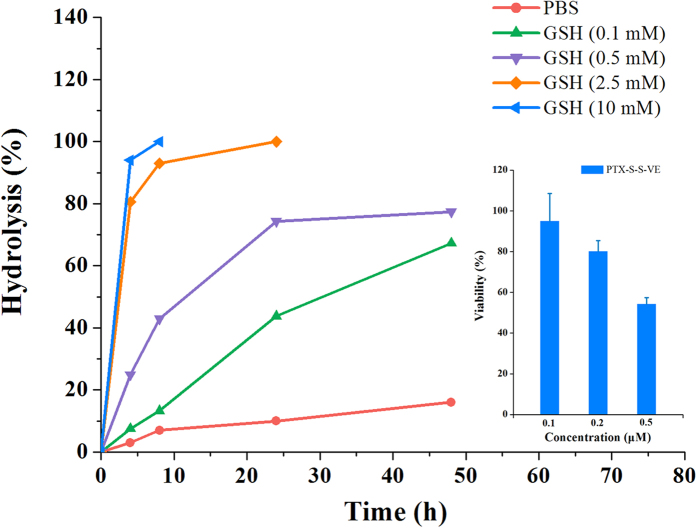
*In vitro* hydrolytic profiles and cytotoxicity against KB-3-1 cell line for PTX-S-S-VE (data are expressed as the mean ± SD, n = 5 wells for each formulation at each concentration). These studies were carried out at 37 °C.

On the basis of the analysis above and considering the redox nature of the tumor microenvironment^33^, we undertook to synthesize PTX-S-S-VE (PTX conjugated with VE via a disulfide bond) because the sufficient GSH^34^,^35^ in tumor cells will cleave the bridge and promote the hydrolysis to free PTX. As shown in Fig. 4, the hydrolysis rate was accelerated with GSH concentration increased. In addition, compared with PTX-VE, the *in vitro* hydrolysis was improved and its anticancer activity was increased.

### Preparation, characterization and storage stability of the NES

**Figure 5:**
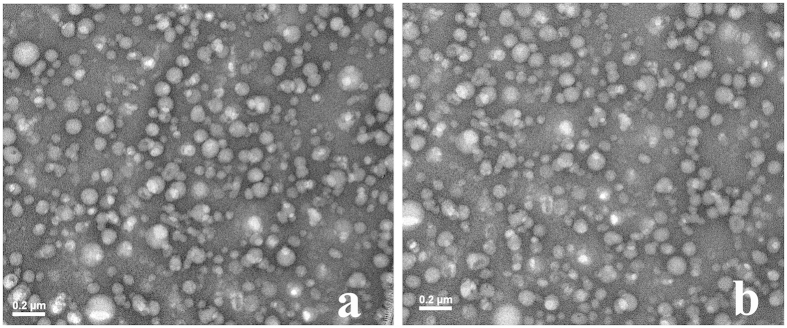
TEM images of PTX-VE/VE/VE_2_-PEG_2000_/water (a) and PTX-S-S-VE/VE/VE_2_-PEG_2000_/water (b) NES.

PTX-VE/VE/VE_2_-PEG_2000_ NES has been prepared because of favorable affinity for PTX-VE, VE, and VE_2_-PEG_2000_. In this study, PTX-S-S-VE/VE/VE_2_-PEG_2000_ NES was also prepared successfully. The particle size, zeta potential, and morphology were characterized. As shown in Fig. 5, for both NES, the particles were of similar spherical shape and size (100–150 nm). Consistent with the TEM images, the dynamic light scattering results showed that both NES were about 130 nm (Table 1, and Figure S8–9). The small PDI indicated that the particles were of a narrow size range. It was demonstrated that the PTX-S-S-VE/VE/VE_2_-PEG_2000_ NES was physically stable for at least 12 months (Table S1). Therefore, the PTX-S-S-VE/VE/VE_2_-PEG_2000_ NES was suitable for *in vivo* studies.

### Tissue distribution

**Figure 6:**
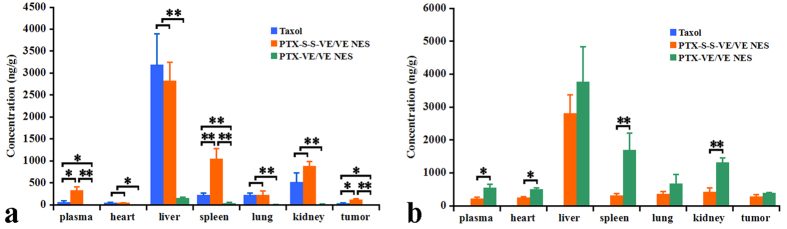
Tissue distribution of parent drug (**a**) and prodrugs (**b**) at 4 h after intravenous administration of Taxol®, PTX-VE/VE/VE2-PEG_2000_/water and water NES PTX-S-S-VE/VE/VE2-PEG2000/ at a PTX equivalent single dose of 20 mg/kg at 4 h (p < 0.05 [*] and p < 0.01 [**]).

To evaluate the *in vivo* performance, the CD1 mice, in the biodistribution study, were administrated intravenously a single dose equivalent of 20 mg/kg PTX. The mice were sacrificed at 4 h and the tissues were harvested. The biodistribution of free PTX and PTX prodrugs are shown in Fig. 6a,b, respectively. For PTX-VE NES, free PTX was only found in the liver, with only a small amount being found in other tissues. This was because of the low cleavage rate of the ester bond, and indicated similar *in vivo* hydrolytic behavior to that seen *in vitro*. On the other hand, PTX-VE NES showed high prodrug accumulation, including higher than that of PTX-S-S-VE NES, with liver (3794.0 ± 1241.6 ng/g) being the major distribution tissue followed by spleen (1753.1 ± 666.7 ng/g) and then kidney (1351.4 ± 179.3 ng/g). The reason for this could be attributed to matching properties of VE oil and the VE group in PTX-VE, as well as PTX-VE being less susceptible to hydrolysis than PTX-S-S-VE. Importantly, it exhibited comparable prodrug distribution in tumors for PTX-VE NES and PTX-S-S-VE NES. In the case of PTX-S-S-VE NES, greater free PTX accumulation was seen than for Taxol^®^ in most tissues. The distribution was statistically higher than Taxol^®^ in plasma (*p *< 0.05), liver (*p *< 0.05), spleen (*p* < 0.05), kidney (*p *< 0.05) and tumor (*p *< 0.05, Student’s *t*-test, paired, two sided). This could be because PTX-S-S-VE and VE are molecularly matched resulting in longer circulation of the PTX-S-S-VE NES in the blood and hence longer *in vivo* retention time than for the Taxol^®^. In addition, the GSH in blood, despite being of much lower concentration than in the tumor, could start the hydrolytic program to some extent, leading to enhanced hydrolysis. The *in vivo* distribution results indirectly reflect the hydrolytic properties of the prodrugs.

### Tumor growth inhibition

**Figure 7:**
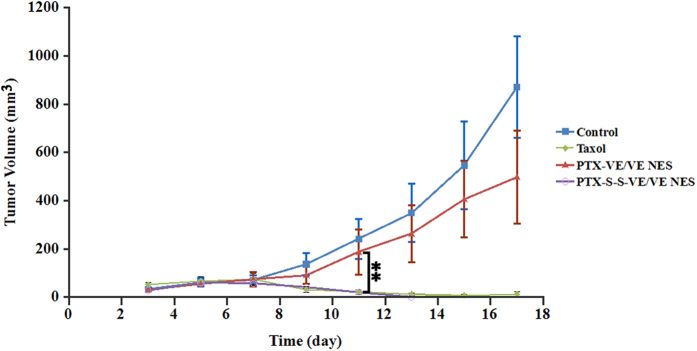
Inhibition of tumor growth by Taxol®, PTX-VE/VE/VE2-PEG2000/water and PTX-S-S-VE/VE/VE2-PEG2000/water NES in KB-3-1 cell subcutaneous xenografts. The formulations were administered intravenously at an equivalent dose of 5 mg/kg of PTX every second day for 5 injections in total (data are expressed as the mean ± SD, n = 5 mice for each formulation) (p < 0.05 [*] and p < 0.01 [**]).

To evaluate the *in vivo* therapeutic effect of the formulations, tumor growth inhibition was studied in a KB-3-1 cells subcutaneous model in Balb/C nude mice. As shown in Fig. 7, the control group showed very rapid tumor growth. There was no significant reduction of the tumor volume in mice treated with PTX-VE/VE/VE_2_-PEG_2000_ NES (*p* > 0.05, Student’s t-test, paired, two sided). However, the mean tumor volume was slightly smaller than for the controls. Not surprisingly, the greatest antitumor activity was observed when the mice were treated with PTX-S-S-VE/VE/VE_2_-PEG_2000_ NES. For this formulation, the tumors were diminished before the fifth injection. Similarly, Taxol^®^ also showed a strong tumor inhibition effect and only one mouse was found to still bear the tumor by the 15^th^ day. In addition, no weight loss occurred in all groups (Fig. S10).

### *In vivo* toxicity

#### Histopathological examination

In order to determine if the accumulation of the formulations is able to damage normal tissues, the histopathological of the heart, liver, spleen, lung, and kidney for each treatment group was examined. The results were shown in Fig. S11. Lesions were not found in the PTX-VE/VE and PTX-S-S-VE/VE NES treated group. However, it is clear that the kidney, in Taxol^®^ treated group, suffers from dilatation and congestion for renal interstitial vascular.

#### Measurement of serum level

AST and ALT are two tests usually used to evaluate the main liver function. In this study, the ALT and AST were significantly elevated in the group treated with Taxol^®^ but not with the PTX-S-S-VE/VE/VE_2_-PEG_2000_/water NES (Table 2), indicating PTX-S-S-VE/VE/VE_2_-PEG_2000_ NES is much better tolerated than Taxol^®^.

#### Hemolysis study

**Figure 8:**
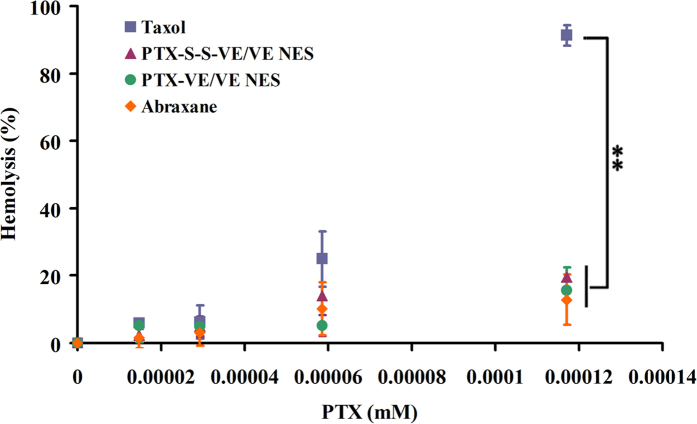
Hemolytic activity of Taxol®, PTX-VE/VE/VE2-PEG2000/water NES, PTX-S-S-VE/VE/VE2-PEG2000/water NES, and Abraxane® at 37 °C for 1 h (data are expressed as the mean ± SD, n = 3 mice for each measurement) (p < 0.05 [*] and p < 0.01 [**]).

It is acknowledged that hemolysis is a major drawback for surfactant based formulations, such as Taxol^®^. In this study, TPGS and VE_2_-PEG, which can each also cause hemolysis, were used for stabilizing the NES. Therefore, to evaluate the safety of the two NES, we studied the hemolysis effect by incubating erythrocytes with the formulations (Taxol^®^, PTX-VE/VE/VE_2_-PEG_2000_ NES, PTX-S-S-VE/VE/VE_2_-PEG_2000_ NES, and Abraxane®) for 1 h at different PTX concentrations. Figure 8 shows hemolysis results for the formulations at various concentrations. At 0.0293 μM of PTX, all formulations were nonhemolytic. As the concentration was increased, hemolysis appeared. At the highest concentration tested (0.117 μM), 91.3% of the erythrocytes were hemolyzed for the Taxol^®^ group, while only 19.5%, 15.6%, and 12.9% of erythrocytes were hemolyzed for PTX-S-S-VE/VE/VE_2_-PEG_2000_ NES, PTX-VE/VE/VE_2_-PEG_2000_ NES, and Abraxane®, respectively. Therefore, it can be suggested that both of the prodrug NES formulations are safer than Taxol^®^ in terms of membrane toxicity.

## Material and Methods

### Materials

PTX was purchased from Lc Laboratories (Woburn, MA). PTX injection (Taxol^®^) was manufactured by Ben Venue laboratories, Inc. (Bedford, OH). TPGS was purchased from Eastman Chemical Company (Anglesey, U.K.). (+)-a-Tocopherol (VE), D-a-Tocopherol succinate, N, N-dicyclohexylurea tert-butyl-2-aminoethylcarbamate, trifluoroacetic acid, tert-butyl methyl ether were purchased from Sigma-Aldrich Corporation (St. Louis, MO).

### Synthesis of PTX-VE, PTX-SA, PTX-SEE, and PTX-S-S-VE

#### Synthesis of PTX-VE

PTX (100.0 mg, 0.117 mmol) was reacted with D-α-tocopherol succinate (62.0 mg, 0.117 mmol) in the presence of N, N’-dicyclohexylcarbodiimide (48.2 mg, 0.234 mmol) and a catalytic amount of dimethylaminopyridine (DMAP) in anhydrous dichloromethane (8 mL) at room temperature under nitrogen atmosphere for 7 h. The resulting mixture was filtered to remove N, N-dicyclohexylurea (DCU) and the filtrate was dried under vacuum. The residue was purified using silica gel column chromatography, eluting with a chloroform-methanol solution with gradually increasing methanol content. The eluting solvent was removed under vacuum to give 95.9 mg of PTX-VE conjugate with the total yield of 60%. The purity was 99.2%, analyzed by the HPLC method.

#### Synthesis of PTX-SA

A solution of PTX (200.0 mg, 0.234 mmol), succinic anhydride (46.9 mg, 0.468 mmol), a catalytic amount of DMAP, and DCM (5 mL) was stirred for 3 h at room temperature. The product was purified using silica gel column chromatography, eluting with a solution of chloroform and methanol (200:1, V/V) to obtain the target compound PTX-SA as white solid (145.0 mg, yield: 65.0%). The purity was 98.8%, analyzed by the HPLC method.

#### Synthesis of PTX-SEE

Monomethyl succinate (34.0 mg, 0.257 mmol) and dichloromethane (5 mL) were stirred in an ice water bath. DCC (53 mg, 0.257 mmol) and a catalytic amount of DMAP were added. The solution turned to suspension. After 5 min, PTX (200.0 mg, 0.234 mmol) was added. After 2 h, the suspension was filtered to remove DCU and then purified by silica gel column chromatography. The elution solution was chloroform and methanol (200:1, V/V) and the target compound was obtained as a white solid (110 mg, yield: 44.5%). The purity was 99.0%, analyzed by the HPLC method.

#### Synthesis of PTX-S-S-VE

A solution of dithiodiglycolic acid (2 g, 11.0 mmol) and anhydrous acetic anhydride (30 mL) was stirred at 30 °C under nitrogen atmosphere for 2 h. Acetic anhydride was removed at room temperature under high vacuum with the addition of toluene three times. The residue was added to a mixed solution of dichloromethane (20 mL), VE (1.0 g, 2.3 mmol) and a catalytic amount of DMAP, and stirred for 5 min at room temperature. The product was purified by silica gel column chromatography, eluting with a solution of hexane, ethyl acetate and acetic acid. The eluting solvent was removed under vacuum to give 0.757 g of acid VE-S-S-COOH with a yield of 55.3%. The acid (0.5 g, 0.84 mmol) and dichloromethane (10 mL) were stirred in an ice water bath. DCC (0.198 g, 1.0 mmol) and DMAP (13.8 mg, 0.01 mmol) were added. The solution changed to a suspension. After 5 min, PTX (0.72 g, 0.84 mmol) was added and the mixture was stirred for a further 2 h. After filtration to remove DCU, the solution was purified by silica gel column chromatography, eluting with a solution of chloroform and methanol (200:1) to obtain the target compound PTX-S-S-VE as white solid (0.814 g, yield: 67.7% ). The purity was 99.4%, analyzed by the HPLC method.

### HPLC analysis

The HPLC system was equipped with a Waters 2487 Dual λ Absorption Detector and a Waters 600 pump (Waters Corporation, Milford, US). The separation was carried out on an ODS HYPERSIL (3 μm, 2.1 mm × 100 mm) column (Thermo Scientific, USA) at a flow rate of 0.5 mL/min. Compound concentration was determined at a ultraviolet wavelength of 228 nm and the injection volume was 20 μL. Gradient elution was applied with a mobile phase of acetonitrile (ACN) and water. The initial mobile phase composition was 40% ACN from 0–36 min, and a linear gradient was applied to reach a composition of 100% ACN after a further 1 min. This was maintained for 30 min and then returned to initial conditions within a further 1 min and equilibrated for 9 min. Total run time was 77 min.

To analyze the hydrolysis of the prodrugs (PTX-VE and PTX-S-S-VE), the initial mobile phase composition (40% ACN) was maintained from 0–12 min, then a linear gradient was applied to reach a composition of 100% ACN after a further 1 min. This was maintained for 23 min and then returned to initial conditions within a further 1 min.

### Hydrolysis

Hydrolytic studies were carried out at 37 °C in pH 7.4 PBS containing 7% DMSO and 0.1% Tween 80. The prodrugs (PTX-SA, PTX-SEE, PTX-VE, PTX-Br_2_-C_16_ and PTX-S-S-VE), equivalent to 11.7 μM of PTX, were added to 100 mL of the medium and placed in a shaking table at a rotating rate of 100 rpm/min. At predetermined time intervals (4, 8, 24 and 48 h), 1 mL of the samples were drawn from the medium and filtered through acetate membranes (0.22 μm, GE Water & Process Technologies, Trevose, PA). The filtrate was mixed with an equal volume of acetonitrile and analyzed using the HPLC method described earlier. The chromatograms were presented as Fig. S7.

### Cytotoxicity

MTS, 3-(4,5-dimethylthiazol-2-yl)-5-(3-car-boxymethoxyphenyl)-2-(4-sulfophenyl)-2H- tetrazolium, inner salt, was used to study the cytotoxicity of the prodrugs following the manufacture’s protocol. Generally, the KB-3-1 cells were seeded in 96 wells at a density of 1 × 10^4^ cells per well. After incubation for 24 h, the cells were treated with PTX, PTX-SA, PTX-SEE, PTX-VE, or PTX-S-S-VE at various concentrations for 48 h. Sterile water treated wells were used as controls. After the incubation, the media was replaced with a mixture of fresh cell culture medium and MTS-based CellTiter 96^®^ AQueous One Solution Cell Proliferation Assay Reagent (Promega Corporation, Madison, WI). The plate was then incubated at 37 °C for a further 2 h. Absorbance was determined at 480 nm using a plate reader. Cell viability was calculated as OD_test_/OD_control_ × 100%.

### Preparation, characterization and storage stability study of the NES

PTX-VE, PTX-S-S-VE, VE, TPGS and VE_2_-TPGS were dissolved in chloroform. PTX-VE (0.70 μM) (or PTX-S-S-VE [0.70 μM]), VE (20 μM), TPGS (1.8 μM) and VE_2_-TPGS (0.34 μM) were added to an Eppendorf Safe Lock Tube^TM^ (1.5 mL) (Next Advance Inc., Averill Park, NY) and the chloroform removed under nitrogen flow and further by vacuum pumping. Water (600 μL) was then added. The mixture was probe sonicated for about 1 min, and the initial NES were mixed with 0.5 g of beads (one third × 1.0 mm in diameter and two thirds × 0.5 mm in diameter). The NES were placed in a BBY24M Bullet Blender Storm (Next Advance Inc., Averill Park, NY) for homogenization.

The particle size and zeta potential of the NES were determined using a Malvern ZetaSizer^®^ Nano ZS (Westborough, MA) three times. In addition, the morphology was observed with a JEOL 100CX II TEM (Tokyo, Japan).

The PTX-S-S-VE/VE/VE2-PEG2000/water NES was stored at 25 °C for 12 months. At predetermined time intervals, samples were withdrawn and evaluated, including the physical appearance, particle size, polydispersity index (PDI), and encapsulation efficiency (EE). These studies were performed in triplicate and data were expressed as mean ± SD.

### Animal studies

All animal research work was approved by the University of North Carolina at Chapel Hill’s Institutional Animal Care and Use Committee and were performed in accordance with relevant guidelines and regulation.

### Biodistribution

Balb/C nude mice (6–8 weeks) were used to evaluate the *in vivo* biodistribution of the formulations. The xenograft models were established by subcutaneous injection of KB-3-1 (5 × 10^6^) cells into the right flanks of the mice. Fifteen mice were randomly divided into three groups (n = 5 for each group) to receive Taxol^®^, PTX-VE/VE/VE_2_-PEG_2000_/water or PTX-S-S-VE/VE/VE_2_-PEG_2000_/water NES, respectively. The formulations were administered intravenously via the tail vein at a single dose of 20 mg/kg PTX equivalent. After 4 h, the mice were sacrificed, and the plasma, heart, liver, spleen, lungs, kidney, and tumor were collected. The tissues were homogenized with 300 μL of HPLC initial mobile phase (ACN:water = 40:60, w/w) using the BBY24M Bullet Blender Storm. The supernatant was obtained by centrifuging the tubes. The PTX and/or prodrugs (PTX-VE and PTX-S-S-VE) were extracted using methyl tert-butyl ether, with PTX-SEE (11.7 μM) as internal standard. The organic phase was transferred to a glass tube and evaporated under nitrogen flow. The residues were redispersed using the initial mobile phase 40:60 (ACN:water, v/v). Analysis was carried out by the HPLC method described earlier.

### *In vivo* anticancer effect

The KB-3-1 tumor bearing nude mice were randomly divided into four groups (n = 5 for each group) to intravenously receive PTX equivalent doses (5 mg/kg) of Taxol®, PTX-VE NES, PTX-S-S-VE NES or physiological saline. The treatments were started on the third day after inoculation, and tumor sizes and body weights were monitored every second day. Tumor volume was calculated as V = 0.5×(L × W^2^), where V represents tumor volume, L the larger perpendicular diameter and W the smaller one.

### Histopathological examination

The histopathological examinations were conducted on the organs of the sacrificed mice. The tissues, including heart, liver, spleen, lung, and kidney, were collected, rinsed with normal saline, fixed in 10% formalin, and then stained with hematoxylin and eosin. Finally, the sections were observed under a microscope for histopathological evaluations.

### Measurement of serum level

Ten CD1 mice were used for the analysis of serum biochemistry. The mice were randomly divided into two groups (n = 5 for each group) to receive Taxol^®^ and PTX-S-S-VE/VE/VE_2_-PEG_2000_/water NES, respectively. The blood samples were collected by enucleating the eyeball after mice received 5 times 10 mg/kg PTX equivalent every second day, and the serum was obtained by coagulating and then centrifuging. Subsequently, the serum samples were analyzed using an AU640 blood biochemical analyzer (Olympus Co., Tokyo, Japan). The serum biochemistry analysis involved the determination of aspartate aminotransferase (AST), alanine transaminase (ALT), and blood urea nitrogen (BUN).

### Hemolysis

To test the potential toxicity of the NES, hemolysis was evaluated. Blank blood was obtained by orbital collection from the CD1 mice. Erythrocytes were immediately obtained by centrifuging the whole blood and washing twice with PBS (pH 7.4) to remove plasma and serum. The isolated erythrocytes (3 × 10^10^ cells) were resuspended and incubated with the formulations at various concentrations at 37 °C for 1 h. The samples were then centrifuged for 10 min at 16000 *g*, and 100 μL supernatants were diluted with an equal volume of PBS and measured for optical density (OD) at 570 nm. A complete hemolytic sample, which was used as a control, was prepared by sonicating blood for 3 min. The percentage of hemolysis was calculated as (OD_test _– OD_formulation_) /OD_control_ × 100%^36^.

### Statistical analysis

All data were expressed as mean ± SD and were analyzed statistically using a one-way analysis of variance (ANOVA) or a two tailed Student’s *t*-test. The differences were considered statistically significant if the *p* value was less than 0.05 (*p* < 0.05).

## Conclusion

In this study, by giving a comparison of hydrolytic release and cytotoxicity for the three conjugates (PTX-VE, PTX-SA, and PTX-SEE), we have found that polarity and steric hindrance are two major factors influencing anticancer activity. Thus, we have designed a programmably hydrolyzable PTX prodrug, PTX-S-S-VE, which undergoes two subsequent hydrolysis steps and releases active drug. Like the previous PTX-VE, PTX-S-S-VE can also be encapsulated in VE/VE_2_-PEG_2000_/water NES for intravenous administration. The *in vivo* distribution for the two NES formulations varied a lot. In nude mice, PTX-S-S-VE/VE/VE_2_-PEG_2000_/water NES had better anticancer activity against KB-3-1 cell line tumors than PTX-VE/VE/VE_2_-PEG_2000_/water NES. Both the hemolysis activity was reduced compared with Taxol^®^. In conclusion, the programmably hydrolyzable PTX prodrug that we have produced demonstrates a bright future for controlling drug release in the treatment of cancer.

## Additional Information

**How to cite this article**: Fu, Q. *et al.* Programmed Hydrolysis in Designing Paclitaxel Prodrug for Nanocarrier Assembly. *Sci. Rep.* **5**, 12023; doi: 10.1038/srep12023 (2015).

## Supplementary Material

Supplementary Information

## Figures and Tables

**Figure 1:**
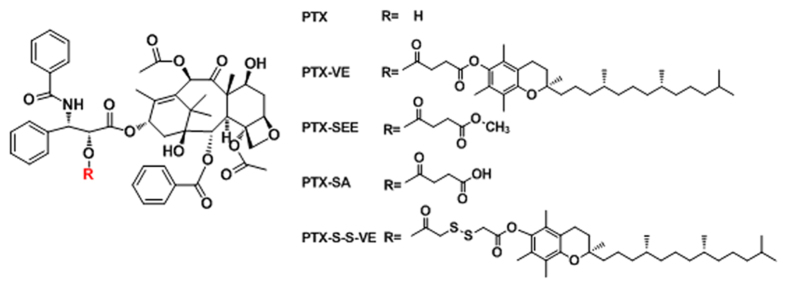
Chemical structures of PTX, PTX-VE, PTX-SEE, PTX-SA, and PTX-S-S-VE.

**Figure 2:**
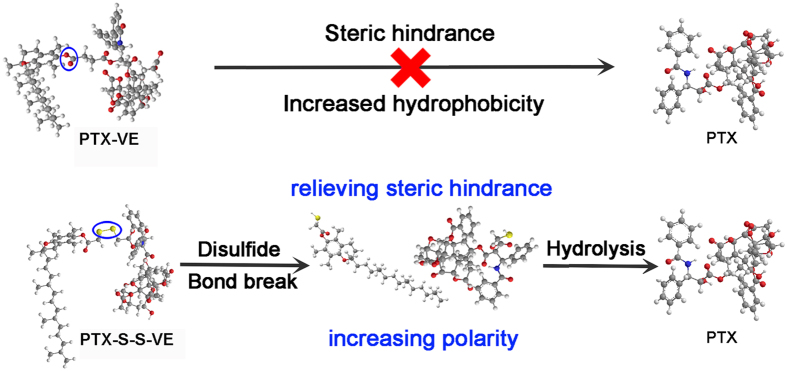
Schematic illustration for the enhanced hydrolysis of PTX-S-S-VE. The prodrug will experience a two stepped hydrolysis to overcome the steric hindrance and polarity hurdles. The disulfide bond will be cleaved in tumor cells because the concentration of GSH in tumor cells is much higher than in blood plasma. This initial hydrolysate will then be further hydrolyzed to PTX because of the increased polarity.

**Table 1:** Particle sizes and zeta-potentials of PTX-VE/VE and PTX-S-S-VE/VE NES (data are expressed as the mean ± SD, n = 3 for each measurement).

**Formulation**	**Z-average (nm)**	**PDI**	**Zeta-potential (mV)**
PTX-VE NES	134.6 ± 0.6	0.175 ± 0.012	–4.03 ± 0.27
PTX-S-S-VE NES	128.1 ± 0.4	0.164 ± 0.027	–3.99 ± 0.26

**Table 2:** Kidney and liver function parameters (AST, ALT, and BUN) in the different experimental group. Values are expressed as mean ± SD.

	**AST(U/L)**	**ALT(U/L)**	**BUN(mg/dL)**
Taxol^®^	398.6 ± 88.7	140.2 ± 44.5	21.8 ± 2.2
PTX-VE NEs	198.0 ± 35.7*	35.2 ± 6.3*	24.2 ± 1.3
Normal Range	54–298	17–77	8–33

*p < 0.05 versus Taxol^®^ as a control.
