# Supplementary material for: Programmed Hydrolysis in Designing Paclitaxel Prodrug for Nanocarrier Assembly
Source: Sci Rep. 2015 Jul 13;5:12023. doi: 10.1038/srep12023 (PMC4499798; doi:10.1038/srep12023)
Supplement: Supplementary Information [file srep12023-s1.pdf]

Supplementary Material for

Programmed Hydrolysis in Designing Paclitaxel Prodrug for Nanocarrier Assembly

Fu Q.[1], Wang Y.[1,*], Ma Y.[4], Zhang D.[1], Fallon J.[3], Yang X. [1], Liu D.[2,*], He Z.[1], Liu F.[3,ϛ]

Affiliations:

[1]School of Pharmacy, and [2]Key Laboratory of Structure-Based Drug Design and Discovery, Ministry of Education, Shenyang Pharmaceutical University, Shenyang 110016, China

[3]Division of Molecular Pharmaceutics, Eshelman School of Pharmacy, University of North Carolina at Chapel Hill, Chapel Hill, NC 27599, USA.

[4]School of Chinese Materia Medica, Guangzhou University of Chinese Medicine, Guangzhou 510006, China.

***Corresponding Authors: Yongjun Wang** Ph.D. and **Dan Liu** Ph.D.

Phone: +86-24-23986325. Fax: +86-24-23986325

E-mail: (Y.W.) i_maple@163.com, (D.L.) sammyld@163.com

ϛ Deceased

≅ This paper is dedicated to the memory of Professor Feng Liu, PhD, 1955-2014 University of North Carolina at Chapel Hill

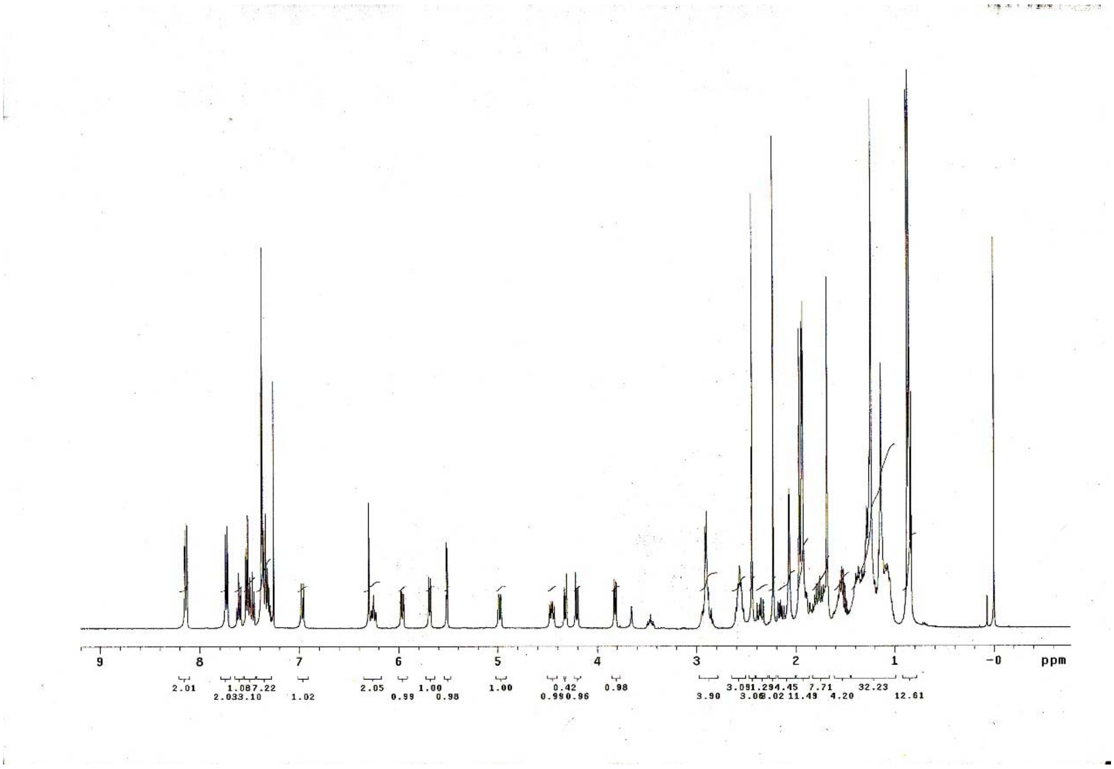

Fig. S1. ^{1}H NMR (400 MHz) spectrum of PTX-Ve in D_2O at 25°C.

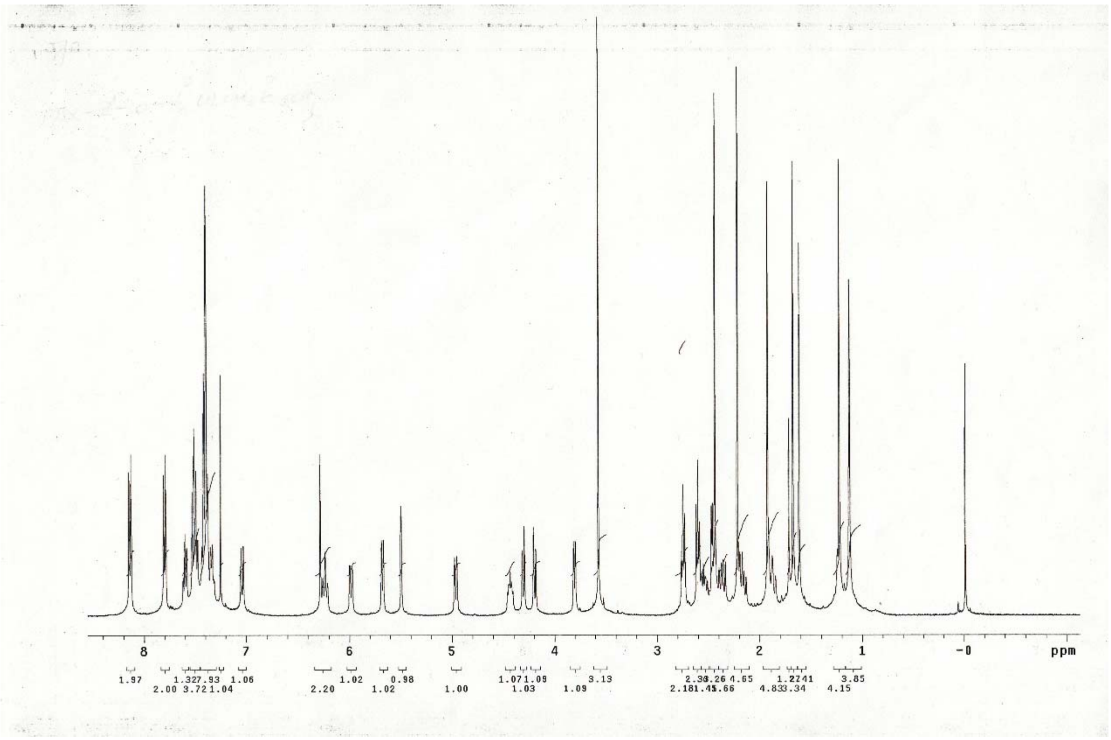

Fig. S2. ^{1}H NMR (400 MHz) spectrum of PTX-SEE in D_2O at 25°C.

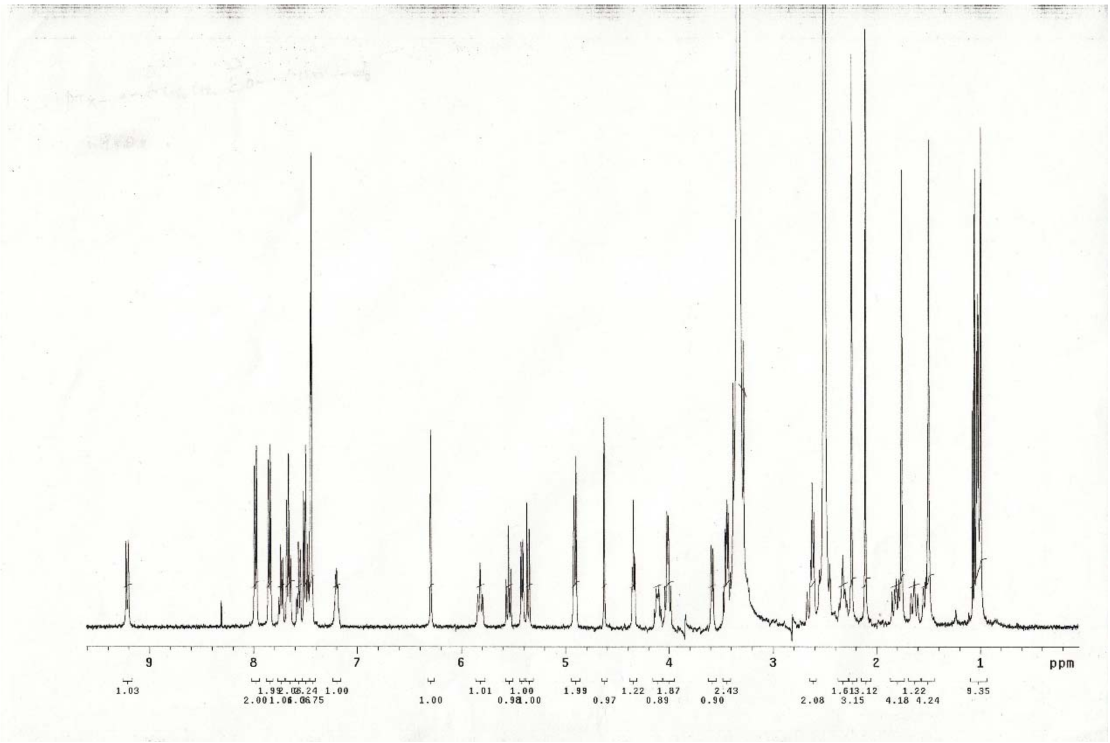

Fig. S3. ^{1}H NMR (400 MHz) spectrum of PTX-SA in D_2O at 25°C.

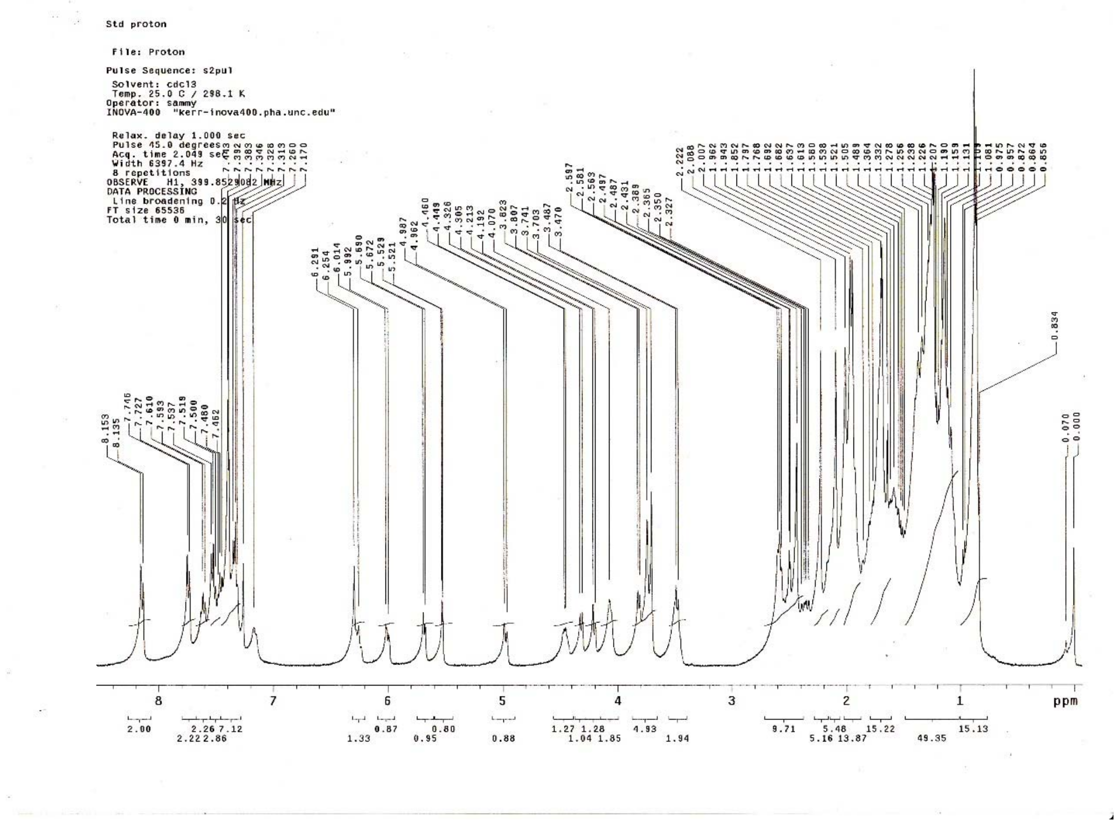

Fig. S4. ^{1}H NMR (400 MHz) spectrum of PTX-S-S-Ve in D2O at 25°C.

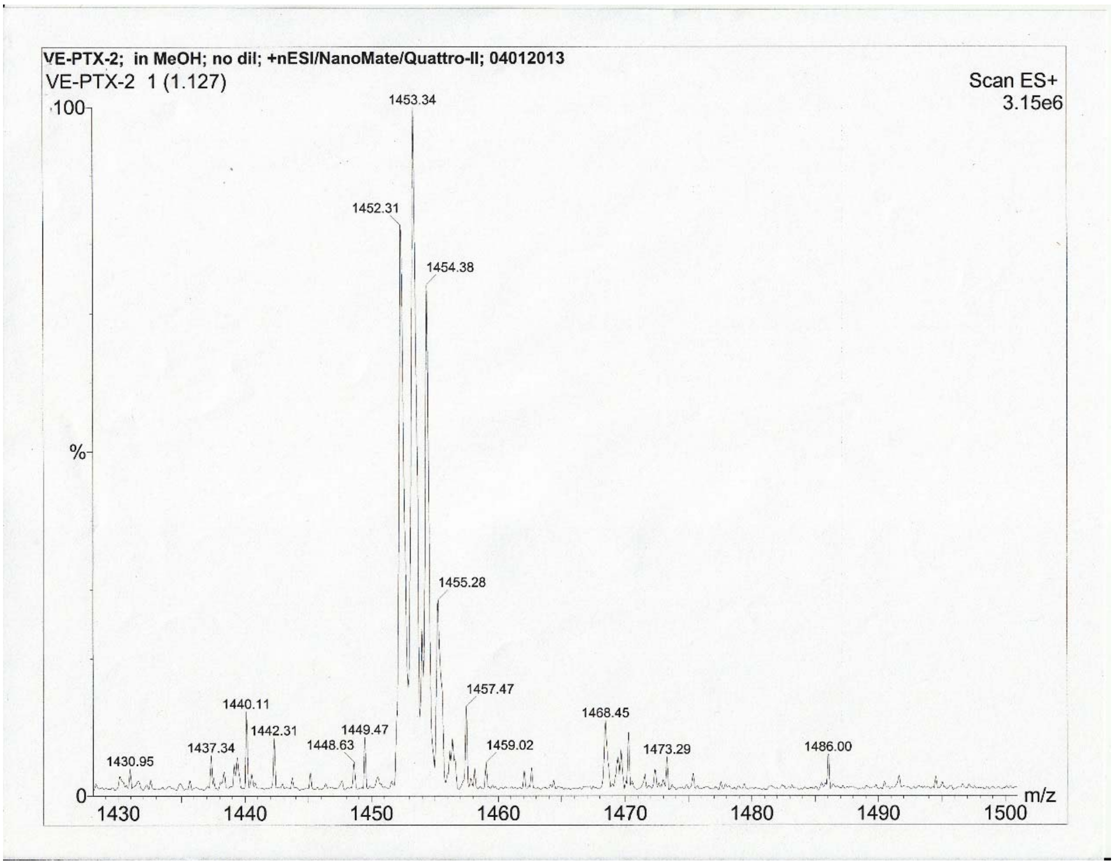

Fig. S5. ESI mass spectrum of PTX-S-S-Ve.

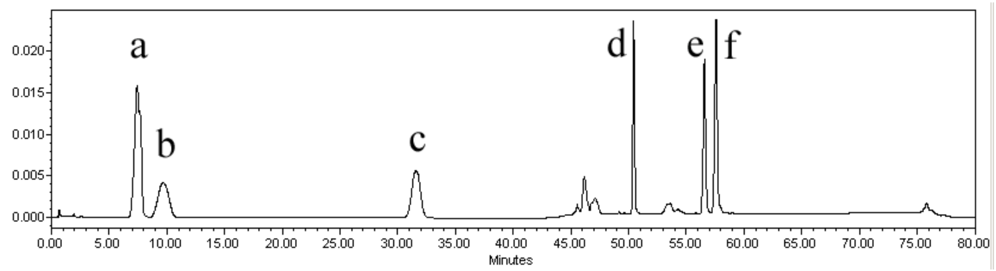

Fig. S6. Chromatogram for PTX and its prodrugs. The retention time was 6.70, 9.29, 31.67, 50.98, 57.72 and 58.83 min for PTX-SA (a), PTX (b), PTX-SEE (c), PTX-C16-Br2 (d), PTX-Ve (e), and PTX-S-S-Ve (f), respectively.

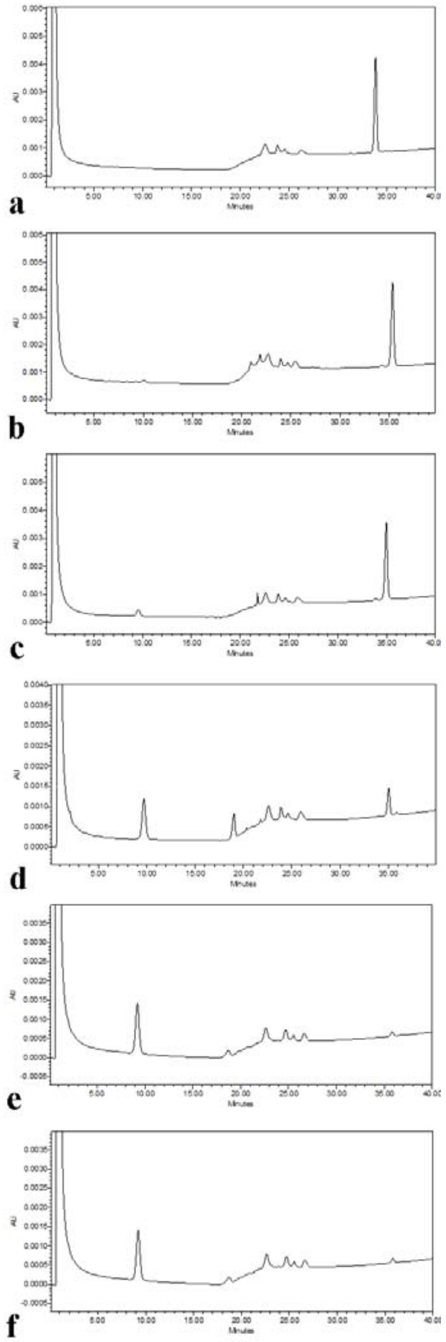

Fig. S7. Chromatograms for PTX-VE (a), and PTX-S-S-VE after the treatment of glutathione at 0 h (b), 4 h (c), 8 h (d), 24 h (e), and 48 h (f) below. The retention time was 9.3, 34.0, and 35.2 min for PTX, PTX-VE, and PTX-S-S-VE, respectively.

Sample Name: PTX-Ve emulsion 1

SOP Name: mansettings.nano

**File Name:**	QIANG FU.dts	**Dispersant Name:**	Water
**Record Number:**	10	**Dispersant RI:**	1.330
**Material RI:**	1.59	**Viscosity (cP):**	0.8872
**Material Absorbtion:**	0.01	**Measurement Date and Time:**	Friday, August 09, 2013 3:17:54 PM

**Temperature (°C):**	25.0	**Duration Used (s):**	60
**Count Rate (kcps):**	486.4	**Measurement Position (mm):**	1.05
**Cell Description:**	Disposable sizing cuvette	**Attenuator:**	4

			**Size (d.nm):**	**% Intensity**	**Width (d.nm):**
**Z-Average (d.nm):**	135.2	**Peak 1:**	164.7	100.0	76.56
**PdI:**	0.161	**Peak 2:**	0.000	0.0	0.000
**Intercept:**	0.919	**Peak 3:**	0.000	0.0	0.000
**Result quality :**	Good				

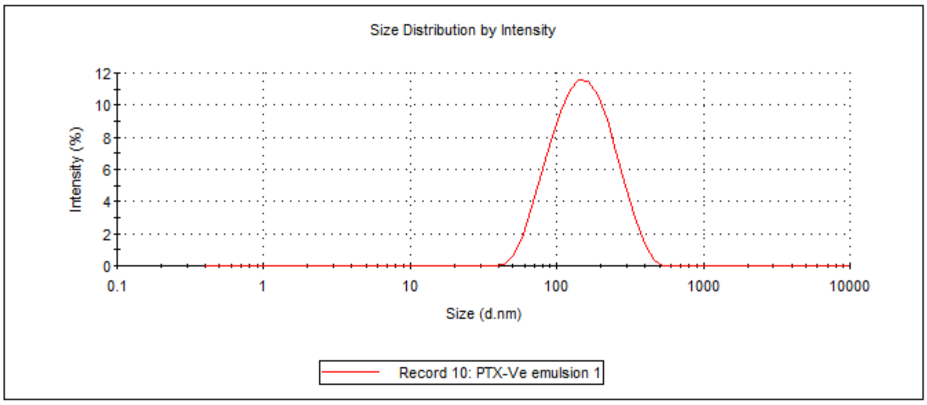

Fig. S8. Particle size distribution for PTX-Ve/Ve NES.

Sample Name: PTX-S-S-Ve emulsion 3

SOP Name: mansettings.nano

File Name: QIANG FU.dts

Record Number: 15

Material RI: 1.59

Material Absorbtion: 0.01

Dispersant Name: Water

Dispersant RI: 1.330

Viscosity (cP): 0.8872

Measurement Date and Time: Friday, August 09, 2013 3:35:54 PM

Temperature (°C): 25.0

Count Rate (kcps): 464.4

Cell Description: Disposable sizing cuvette

Duration Used (s): 60

Measurement Position (mm): 1.05

Attenuator: 4

			Size (d.nm):	% Intensity	Width (d.nm):
**Z-Average (d.nm):**	128.3	**Peak 1:**	151.0	100.0	63.46
**PdI:**	0.138	**Peak 2:**	0.000	0.0	0.000
**Intercept:**	0.923	**Peak 3:**	0.000	0.0	0.000
**Result quality :**	Good				

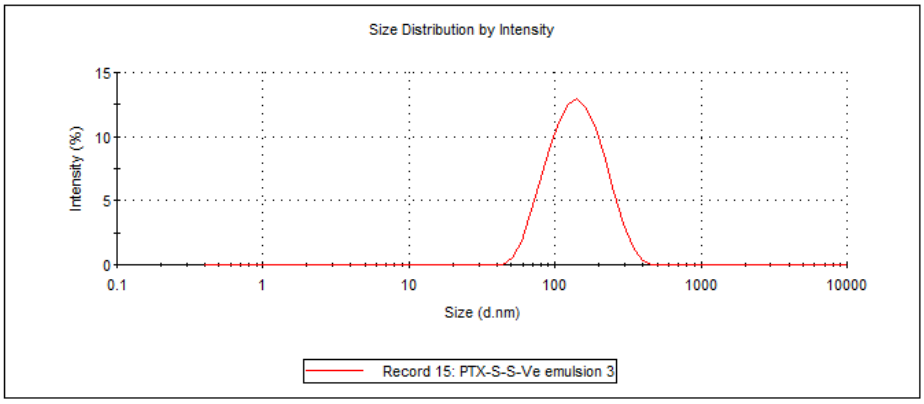

Fig. S9. Particle size distribution for PTX-S-S-Ve/Ve NES.

Table S1. The stability evaluation for PTX-S-S-VE/VE NES

Time (month)	Physical appearance	Z-average (nm)	PDI	EE (%)
0	Good	128.1 ± 0.4	0.175 ± 0.012	98.7 ± 0.3
1	Good	134.6 ± 1.8	0.189 ± 0.021	98.1 ± 0.3
2	Good	129.4 ± 3.2	0.180 ± 0.017	98.9 ± 0.4
3	Good	126.5 ± 0.9	0.174 ± 0.009	98.7 ± 0.3
6	Good	130.9 ± 1.1	0.181 ± 0.011	98.6 ± 0.2
12	Good	134.1 ± 2.0	0.181 ± 0.021	98.1 ± 0.4

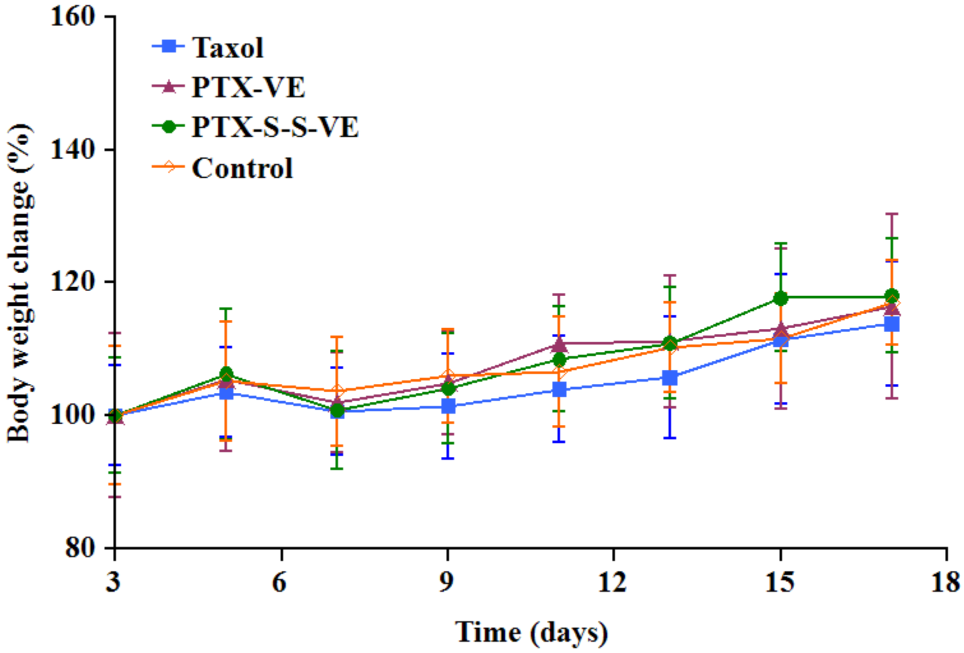

Fig. S10. Body weight change for the nude mice over 18 days after tumor inoculation treated with Taxol®, PTX-VE/VE NES, PTX-S-S-VE/VE NES, and saline. The formulations were administered intravenously at an equivalent dose of 5 mg/kg of PTX every second day for 5 injections in total (data are expressed as the mean ± SD, n=5 mice for each formulation).

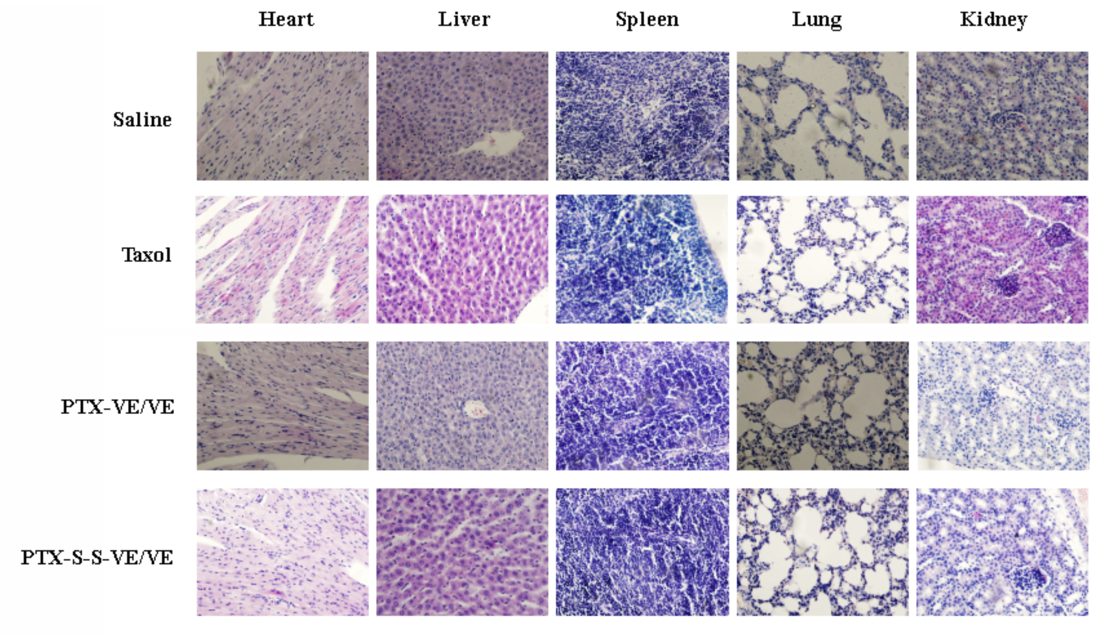

Fig. S11. Typical histopathologic images of hearts, liver, spleen, lung, and kidney after treatment with saline, Taxol®, PTX-VE/VE NES and PTX-S-S-VE/VE NES. The formulations were administered intravenously at an equivalent dose of 5 mg/kg of PTX every second day for 5 injections in total (data are expressed as the mean ± SD, n=5 mice for each formulation).
